# Record of Observations, Etc., with a View of Determining Sex in Utero

**Published:** 1874-09

**Authors:** D. A. K. Steele

**Affiliations:** Resident Physician Cook County Hospital


ARTICLE VI.—*Record of Observations in Fifty Obstetrical Cases, with a View of Determining Sex in Utero.* By D. A. K. STEELE, M.D., Resident Physician Cook County Hospital.

The interesting fact, that in a certain proportion of cases we may determine with some degree of accuracy the sex of the child in utero by auscultating the fœtal heart, has long been established. Some writers have been very enthusiastic in defense of their observations. One author asserts that out of one hundred and nine cases he was correct in one hundred, while Frankenhauser gravely asserts (in the *British Med. Journal*, Aug. 16, 1873) that in fifty cases which he examined. in every instance his diagnosis was *correct.* Other writers, perhaps more conscientious or less careful, have declared that their observations have been comparatively worthless.

Noticing the disparity between statements of different writers on this subject, on the 1st of February, 1874, I determined to make a series of observations with a view of ascertaining in what per cent. of cases our predictions would be verified. Since that time I have kept a careful record of fifty cases, the result of which I give herewith, for convenience, in a tabulated form, giving all the facts of interest as concisely as possible, without wearying the reader with a detailed statement, under the following heads:

1st, Nationality of Mother; 2nd, Age of Mother; 3rd, Duration of Labor; 4th, Weight of Child; 5th, Sex of Child; 6th, Record of Fœtal Heart.

Multipara, 13 cases; Primapara, 37 cases; total, 50.

NATIONALITY OF MOTHER.

| | | | |
|---|---|---|---|
| United States, | 16 | Norway, | 4 |
| Sweden, | 9 | Denmark, | 1 |
| Ireland, | 7 | Switzerland, | 1 |
| Germany, | 6 | Foreign, 68 per cent. | |
| England, | 6 | Native, 32 per cent. | |

AGE OF MOTHER.

| | | |
|---|---|---|
| February, | Maximum, 37 years, Minimum, 18 " | Average, 25 years. |
| March, | Maximum, 37 years, Minimum, 17 " | Average, 25 years. |
| April, | Maximum, 40 years, Minimum, 17 " | Average, 24 years. |
| May, | Maximum, 25 years, Minimum, 17 " | Average, 21 years. |
| June, | Maximum, 32 years, Minimum, 24 " | Average, 27 years. |
| Grand Total, | Maximum, 40 years, Minimum, 16 " | Average, 24 years. |

### DURATION OF LABOR.

| | | |
|---|---|---|
| February, | Maximum, 20 hours,<br>Minimum, 3 " | Average, 13 hours. |
| March, | Maximum, 18 hours,<br>Minimum, 4 " | Average, 11 hours. |
| April, | Maximum, 36 hours,<br>Minimum, 6 " | Average, 13 hours. |
| May, | Maximum, 36 hours,<br>Minimum, 1 " | Average, 11 hours. |
| June, | Maximum, 24 hours,<br>Minimum, 8 " | Average, 13 hours. |
| Grand Total, | Maximum, 36 hours,<br>Minimum, 1 " | Average, 12 hours. |

### WEIGHT OF CHILDREN.

| | | |
|---|---|---|
| February, | Maximum, 10 lbs.<br>Minimum, 6 " | Average, 8¼ lbs. |
| March, | Maximum, 10 lbs.<br>Minimum, 8 " | Average, 8¾ lbs. |
| April, | Maximum, 10 lbs.<br>Minimum, 6 " | Average, 8 lbs. |
| May, | Maximum, 11¼ lbs.<br>Minimum, 4 " | Average, 8 lbs. |
| June, | Maximum, 9½ lbs.<br>Minimum, 5½ " | Average, 8 lbs. |
| Grand Total, | Maximum, 11¼ lbs.<br>Minimum, 4 " | Average, 8 1-5 lbs. |

### SEX OF CHILDREN.

Children born in February—Male, 5, Female, 3 ; total, 8.
Children born in March—Male, 10, Female, 3 ; total, 13.
Children born in April—Male, 8, Female, 5 ; total, 13.
Children born in May—Male, 8, Female, 2 ; total, 10.
Children born in June—Male, 4 ; Female, 2 ; total, 6.
Grand Total—50 ; Male, 35 ; Female, 15.

### RECORD OF FŒTAL HEART.

| | | |
|---|---|---|
| February, | Average Male Fœtal Heart, 138<br>Average Female Fœtal Heart, 137 | Average, 137½. |
| March, | Average Male Fœtal Heart, 128<br>Average Female Fœtal Heart, 139 | Average, 133½. |
| April, | Average Male Fœtal Heart, 131<br>Average Female Fœtal Heart, 140 | Average, 135½. |
| May, | Average Male Fœtal Heart, 127<br>Average Female Fœtal Heart, 125 | Average, 126. |
| June, | Average Male Fœtal Heart, 123<br>Average Female Fœtal Heart, 130 | Average, 126½, |
| Grand Total, | Average Male Fœtal Heart, 129<br>Average Female Fœtal Heart, 136 | Average, 132. |

### PREDICTIONS.

| | | |
|---|---|---|
| Male Fœtal Heart, 35. | Correct,<br>Doubtful,<br>Wrong, | 24.<br>4.<br>7. |
| Female Fœtal Heart, 15. | Correct,<br>Doubtful,<br>Wrong, | 8.<br>3.<br>4. |

From these observations we deduce the following facts :

*First.* In the majority of cases *male* fœtal hearts are slower than *female.*

*Second.* 132 fœtal pulsations per minute is the average dividing line. Below this, $68\frac{4}{7}$ per cent. are male; 20 per cent. female; $11\frac{3}{7}$ per cent. doubtful. Above this, $53\frac{1}{3}$ per cent. are female; $26\frac{2}{3}$ per cent. male; 20 per cent. doubtful.

We may notice in this another demonstration of the fickleness of the female heart.

*Third.* The most accurate observations are made during the last four weeks of gestation.

*Fourth.* The rapidity of the heart's action is increased in proportion to feebleness of the fœtus.

*Fifth.* Calcareous or fatty degeneration of the placenta renders the pulsations feeble and irregular.

*Sixth.* In some cases it would be possible to diagnose diseased conditions of the placenta from careful observation of the fœtal heart.

The sounds of the fœtal heart may be rendered feeble by thick abdominal walls, tense abdominal muscles, or the presence of a large quantity of amniotic fluid.

The attachment of the placenta may be determined by the souffle, a soft blowing murmur, synchronous with the maternal heart.

The weight of the child does not increase the force of the fœtal heart. During labor the fœtal heart becomes accelerated and irregular in its action. The most accurate diagnosis was the result of repeated observations, as the fœtal heart might be accelerated in its action by temporary excitement during a single examination.

The observations were conducted by means of an ordinary Cammana's stethoscope, and my experience has been, that a better sound is heard when the bell of the instrument is moistened and applied to the abdomen without pressure, as the peculiar thrill of the fœtal circulation is lost when the stethoscope is grasped by the fingers.

For convenience I divided the abdomen into four segments, by means of rectangular lines crossing at the centre of the uterus. These I designated as the right and left, upper and lower quarters.

In the 50 cases recorded above, the fœtal heart was heard most distinctly—in 30 cases in left lower quarter; in 12 cases in right lower quarter; in 5 cases in left upper quarter; and in 3 cases in right upper quarter. And it is proper to add, that the last three cases were all breech presentations; all the others being vertex. Forty-one were in the first position, and six in the third.

If the fœtal heart is heard most distinctly above the transverse line, the presentation is most likely breech. If below, almost certainly vertex. Much valuable information can be gained by inspection and palpation in determining the position of the fœtus, and we should not rely entirely on auscultation.

I am indebted to my friend Dr. Strong for many valuable suggestions as to the best method of conducting these observations.

